# The role of national health insurance for achieving UHC in the Philippines: a mixed methods analysis

**DOI:** 10.1080/16549716.2018.1483638

**Published:** 2018-06-19

**Authors:** Konrad Obermann, Matthew Jowett, Soonman Kwon

**Affiliations:** a Mannheim Institute for Public Health (MIPH), Heidelberg University, Mannheim, Germany; b Department of Health Systems Governance & Financing, World Health Organization, Geneva, Switzerland; c School of Public Health, Seoul National University, Gwanak-gu, South Korea

**Keywords:** Universal Health Coverage (UHC), Philippines, social health insurance, PhilHealth, health care financing

## Abstract

**Background**: Achieving Universal Health Coverage (UHC) has by now become a key health policy goal in many countries and some form of National Health Insurance (NHI) is often used for this. The Philippines has had more than 50 years’ experience with social health insurance and in 1995 established PhilHealth, the country’s national health insurer.

**Objectives**: Analyzing the role of the Philippine NHI scheme in moving towards UHC, identifying potential avenues for improvement as well as indicating challenges and areas for further development.

**Methods**: This paper is based on a mixed methods approach including extensive literature search, data from PhilHealth and other sources, and key informant interviews with staff at PhilHealth, health care providers, and policy experts at national and international level.

**Results**: Major achievements were the expansion of population coverage using an earmarked revenue source (‘Sin Tax’), the introduction of the no-balance-billing to prevent co-payments, and the Health Facilities Enhancement Program to improve quality. The share of PhilHealth in total health expenditures is still only 14%, managing quality and cost of providers remains insufficient, the benefit coverage does not reflect the country’s burden of disease, and financial protection for PhilHealth members is low. The UHC bill would provide a massive jump forward as all Filipinos would then be automatically enrolled in and thus entitled to the benefits of PhilHealth.

**Conclusions**: For expanding a contribution-based NHI beyond formal employment there needs to be a large increase in budget transfers to cover for citizens unable to contribute. The Philippine UHC bill shifts from the idea of contribution leading to entitlement to the idea of citizenship leading to entitlement and can thus be seen as a paradigmatic change in thinking about NHI. There are three areas that we believe are of key importance in developing further NHI: (i) governance, (ii) financial impact, and (iii) strategic purchasing.

## Background

Achieving Universal Health Coverage (UHC) has by now become a key health policy goal in many countries throughout the world, clearly reflected in the World Health Assembly Resolution 58.33 (2005) and also in the SDG goal 3.8 [1]. Especially in low- and middle-income countries (LMIC), the concept of UHC – that is, everyone receiving quality health care without suffering financial hardship – has gained wide acceptance [2].

One way of working towards equitable financing of health care is the use of a National Health Insurance (NHI) as the basis for a shift to true universality, which includes a mandatory contribution scheme, pooling at the national level and purchasing a package of services for all citizens, thus reflecting thinking across entire health systems and populations, not only individual schemes [3]. This would also include the issue of continuum of care ranging from preventive measures to acute and long-term care, including palliative care.

The Philippines, after having had the ‘Medicare’ health insurance since 1969, embarked on an ambitious NHI program with the creation of PhilHealth in 1995. A 2006 paper concluded that in many aspects PhilHealth could be seen as a successful and well-functioning organization, but a number of challenges remain [4].

The Philippine government has repeatedly endorsed the use of its NHI as a vehicle for achieving UHC, last in its ‘Philippine Health Agenda 2017–2022’ (Department of Health AO 2016–0038). A major political impetus occurred in September 2017, when the Universal Health Coverage bill 5784 was passed by the House of Representatives in the Philippines [5]. The bill provides a massive jump forward in terms of UHC as it stipulates that all Filipinos are automatically enrolled in, and thus entitled to the benefits of, the National Health Security Program (the new name for the National Health Insurance Program). The Bill covers a wide range of health care issues like governance (including Health Technology Assessment, HTA), regulation, human resources, health service delivery and income retention by hospitals, and a health information system. The Bill distinguishes between members in the formal group (i.e. everyone rendering services in government or private employment, business owner, migrant workers, self-earning individuals), who are obliged to pay a premium, and members in the non-formal group (for whom full funding would be included in the national General Appropriations Act). All senior citizens are also mandatorily covered.

PhilHealth (which is covered in detail in the Bill) will play a decisive role in implementing the legal stipulations of the Bill. (It should be noted here, that so far only the lower house version has passed the Bill. The senate will discuss its version of the Bill and a combined version has to go through three readings before it turns into law.)

This paper analyzes the environment, achievements and challenges for PhilHealth, the National Health Security Program carrier, in moving towards UHC; it includes good practice for inspiration and points towards options for further development.

The remainder of the paper is organized as follows: after describing the background and recent developments concerning PhilHealth and the delivery and financing of health care in the Philippines, the material and methods employed are explained. Next are the findings which briefly describe the organization PhilHealth, and two main sections describing (a) the role and impact of PhilHealth on financing health care, and (b) the role and impact of PhilHealth in service quality improvement. In the discussion section, these findings are critically assessed, data and knowledge gaps are pointed out and the findings are put into an (international) perspective. Finally, the conclusion reflects on the role of a national health insurance scheme in moving towards UHC and suggests three potential areas for further debate and inquiry in improving PhilHealth’s capacity and effectiveness.

The Philippines is an archipelago in South-East Asia with an ethnically diverse population of more than 100 million, scattered over 600 inhabited islands. About half of the population now lives in cities, of which almost half live in slums. The country is in the midst of an epidemiological ‘dual burden’ with morbidity and mortality from maternal, neonatal and infectious causes still prominent, while those based on sedentary lifestyle and chronic conditions are rising. Indicators of health status have steadily improved in the last 50 years, but there is high inequality regarding health outcomes between socio-economic classes and between geographical regions [6]. Despite the country’s economic growth averaging 6.2% during the Aquino administration (2010–16), the Philippines are beset by persistent problems of inequality and poverty with the Gini coefficient standing at 46% (2012) and poverty headcount at 25.2% (2012) [7].

### Recent developments of health care financing and NHI in the Philippines

Although total health care spending (with 4.7% of GDP in 2014) in the Philippines is at par with its neighbors and peers (e.g. Indonesia 2.8%; Malaysia 4.2%; Thailand 4.1%; Vietnam 7.1%; World Health Organization [WHO] Global Health Expenditure Database), access to services for the poor and quality of care remain a challenge. With 54% out-of-pocket spending, the Philippines (Indonesia 47%; Thailand 12%, Vietnam 37%) has one of the highest percentages in the region.

There is a clear and strong political commitment: the Aquino Health Agenda (AHA) had the goal of attaining Kalusugang Pangkalahatan (KP or UHC) by 2016, and so has the Philippine Health Agenda (PHA) 2017–2022 under President Duterte. The Universal Health Coverage Bill 5784 of September 2017 stipulates that all Filipinos are automatically enrolled in and thus entitled to the benefits of the National Health Security Program.

**Figure 1.**
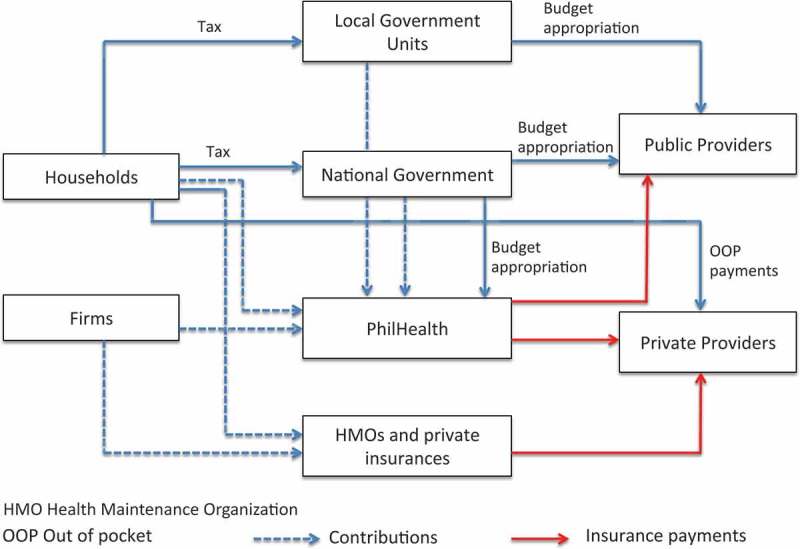
Financial flows. Source: Authors, based on ref [6].

PhilHealth is tasked with working on improving access to quality care and reducing out-of-pocket (OOP) expenses. The corporation is embedded in a complex web of responsibilities, accountability and flow of funds, having to work with national and local governments, politicians, technical institutions, public and private providers, health maintenance organizations, and private firms, amongst others (Figure 1). More details on PhilHealth’s emerging role in this web will be discussed later in the ‘Results’ section.

The introduction of the so-called ‘Sin Tax’ in 2012 (Republic Act RA 10351) was a remarkable feat. Amidst a generally weak tax policy and administration [8], the country reformed the excise tax on tobacco and alcohol, channeling most of its revenues to health. Within the first year, the Sin Tax raised more than 1.2 billion USD, allowing the enrolment of an additional 14 million families or roughly 45 million Filipinos into PhilHealth [9]. Eighty-five percent of revenues are allocated for health, of which 80% are for achieving UHC and 20% for medical assistance and the improvement of health facilities. The Department of Health (DOH) budget for 2016 is more than twofold higher (122.63 billion PHP, 2.44 billion USD) than in 2013, and money earmarked for PhilHealth in the DOH budget went up to 43.89 billion PHP (0.88 billion USD), used mainly to pay the contributions for the indigent members of PhilHealth [10] – an example, on the pathway of progress towards UHC, in which population coverage was greatly expanded using an explicit earmarked revenue source.

Two major initiatives towards improving access to quality care were the introduction of the so-called no-balance-billing (NBB) for government hospitals and the expansion of the Health Facilities Enhancement Program (HFEP) in 2011. The NBB is a policy response to the strategy of many hospitals charging patients on top of what PhilHealth reimburses for the specific condition and treatment. The NBB has now explicitly forbidden government-owned hospitals to charge patients (indigent, sponsored, senior, lifetime members) anything over and above what PhilHealth reimburses for case rates, Z-benefits and primary benefits at all accredited government health care institutions [11] (amendment: https://www.philhealth.gov.ph/circulars/2017/circ2017-0006.pdf). The HFEP, starting in 2007, is a nationwide program by the DOH that aims to improve the supply side and its budget has gone up from 43 million PHP (0.86 million USD; 2007) to 26.9 billion PHP (0.54 billion USD; 2016), in part due to the allocations from the Sin Tax revenues [10].

Textbox: Some key developments in the PhilHealth history

**Table:** 

President	Year	
Ferdinand Marcos (1965–1986)	**1969**	Creation of Medicare, forerunner of PhilHealth; **Coverage: about 20%**
	**1986**	President Marcos toppled
Cory Aquino (1986–1992)	**1992**	Devolution: Shifting budget (about 39%) and responsibilities in the health sector (primary and secondary level services) from central government to LGUs
Fidel Ramos (1992–1998)	**1995**	RA 7875 (National Health Insurance Act): Creation of PhilHealth; policy goal to achieve universal coverage until 2010; **Coverage: just under 50%**
Gloria Macapagal-Arroyo (2001–2010)	**2004**	The ‘Plan 5 Million’ to enrol an additional 5 million members into PhilHealth as part of the election campaign of President Macapagal-Arroyo led to significant increase in PhilHealth membership; **Coverage: about 70%**
	**2009**	Introduction of Philippine DRGs
Benigno Aquino (2010–2016)	**2010**	Aquino Health Agenda 2010–16
	**2011**	Introduction of Z-Benefits covering particularly costly procedures
	**2011**	No-balance-billing (NBB) for government hospitals and the expansion of the Health Facilities Enhancement Program (HFEP)
	**2012**	Introduction of Primary Care Benefits (PCBs) outpatient package
	**2012**	Sin Tax in 2012 (Republic Act RA 10351)
	**2013**	Introducing Tsekap outpatient package for the indigent
	**2013**	Revised Implementing Rules and Regulations of the National Health Insurance Act of 2013 (RA 7875) allowing the implementation of quality assurance standards
	**2013**	Rise in minimum contribution for the IPP from 1200 PHP to 2400 PHP
	**2014**	DOH developed Drug Price Reference Index DPRI
Rodrigo Duterte (since 2016)	**2017**	Philippine Health Agenda 2017–2022; **Coverage: about 90%** Universal Health Coverage Bill 5784 passed by House of Representatives

Despite these highly commendable initiatives and a clear understanding at policy level about the need for different interventions at different parts of the system, overall challenges remain, namely (i) high OOP expenditures, (ii) supply-side constraints and (iii) access barriers, in particular for the poorer parts of the population [11]. What is the role of PhilHealth in addressing these challenges and what is needed for a more targeted response and more impact?

## Methods

We employed a mixed methods approach, i.e. a combination of extensive literature search including grey and unpublished literature (leading to a total number of 97 relevant papers), data from PhilHealth and other sources (a total of 41 files), and interviews with technical and policy staff at PhilHealth (8 interviews), health care providers (5 interviews), and policy experts at the Department of Health (DOH) (2 interviews), in academe (2 interviews) and international organizations (4 interviews). Available country data was gathered and analyzed and put into an international perspective. In particular, key data on coverage, utilization and financial impact of PhilHealth was collated in the form of secondary data analysis. For assessing the impact of PhilHealth at the population level, we used instruments that look at financial protection and utilization of quality services [11, chapter 5].

## Results

### The organization PhilHealth

The structure of PhilHealth is characterized by a combination of headquarters and regional organization. As of the end of 2016, PhilHealth had a total of 6346 staff, of whom 896 work in the headquarters and 5450 work in the 22 PhilHealth Regional Offices (PROs). The PhilHealth president is a political appointee and reports to the president of the country and the board headed by the secretary of health. The total administrative expenses stood (in 2016) at about 7.2% of overall expenses (including benefit payments) of 114.5 billion PHP (2.3 billion USD).

The role of PhilHealth vis-à-vis providers, both governmental and private, is complex. Ever since the devolution of health services to the Local Government Units (LGUs) in 1992, the DOH has had a complex relationship with LGUs, providing them with much-needed technical support, but at the same time not being able to really influence service delivery [11].

**Figure 2.**
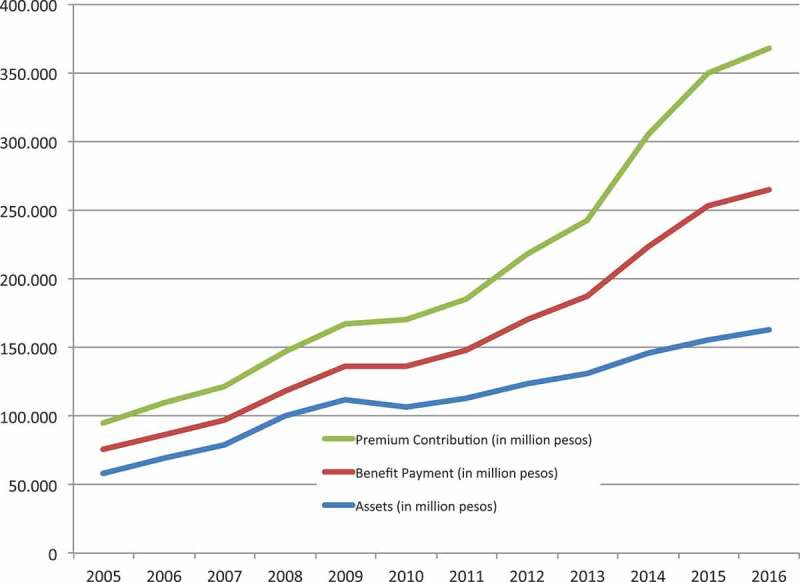
Premium contributions, benefit payments and assets, 2005–16. Source: PhilHealth Corporate Planning.

Figure 2 shows that premium contributions were consistently higher than benefit payments and, consequently, the corporation was able to build up substantial reserves, at present being almost half of total annual contributions. Despite this, rapidly expanding membership (and expenses) might jeopardize these reserves if not accompanied by appropriate contributions from the insured and/or the government. At any rate, it might be appropriate to reflect on the amount of reserves in a public schemes going beyond a relatively low amount to cover cash flow issues, spikes in demand, etc. Holding excessively high reserves when benefits are limited is hard to defend from a UHC perspective.

### The role of PhilHealth in UHC progress

Based on the UHC dimensions of *coverage, benefits* and *financial risk protection* [12], the three dimensions identified by Cashin and colleagues [13] for sustaining progress towards UHC, and Kutzin’s [14] attempt to describe the role of health financing in achieving UHC (and thus fit UHC into the existing health system frameworks), we divided both the following section and the Discussion section into (i) the role and impact of PhilHealth in financing health care and (ii) the role and impact of PhilHealth in service quality and utilization.

#### (I) The role and impact of PhilHealth in financing health care

##### The role of PhilHealth in financing health care

**Figure 3.**
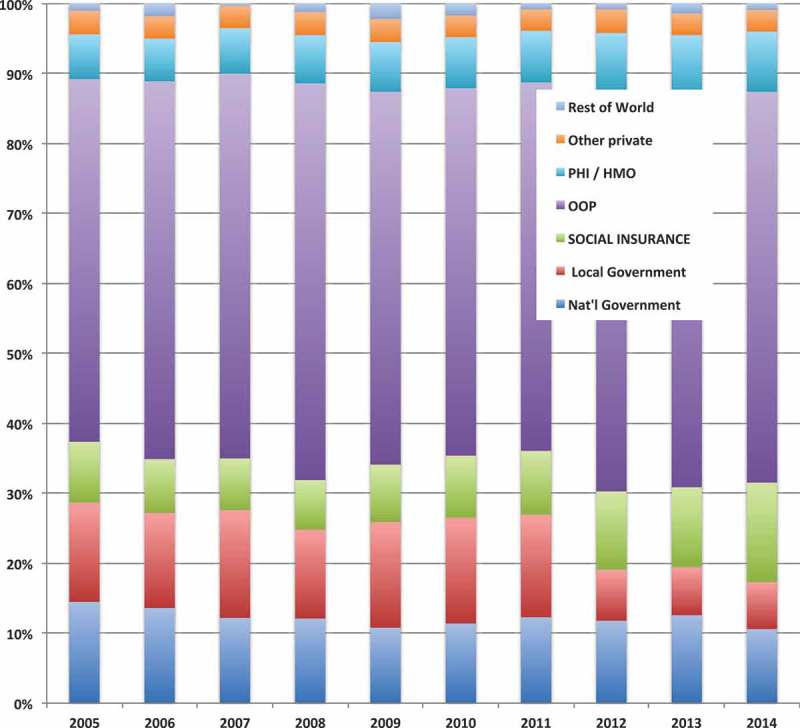
Health expenditure by source of funds, 2005–14. Source: Ref [15], Philippine National Health Accounts.

The 2014 Philippine National Health Accounts (PNHA) show that 4.6% of GDP has been spend on health, totaling 585.3 billion PHP (11.7 billion USD), with a nominal growth of 10.4% from 2013 (at constant 2006 prices, the real growth was about 6%). Per capita health expenditure rose from 5.400 to 5.860 PHP (108 to 117 USD) – an increase of 8.5% (4.2% in constant 2006 prices). Sixty-eight percent of the money came from private sources (mostly out-of-pocket expenses, with 52%), the government contributed 17% (11% national, 6% Local Government Units, LGUs, i.e. Provinces and Municipalities), PhilHealth came third with 14%, and the rest of the world (including development aid) contributed less than 1% (see Figure 3).

This reflects a long-standing trend, which is characterized by substantial economic growth, high one-digit inflation, continuous population growth and a slowly growing share of health spending from 3.9% of GDP (2005) to 4.7% (2014) [15]. PhilHealth’s share of total health expenditure (THE) remains modest because of a low contribution rate (currently at 2.5%) and a low income ceiling for contributions (currently at 35,000 PHP, 700 USD). These shares are in stark contrast with the Health Sector Reform Agenda (HSRA) target of 40% for all government health expenditure and 20% for OOP payments [15]. Figure 3 shows that the impact of the Sin Tax (as seen in the change from 2013 to 2014) was moderate in raising PhilHealth’s share of overall health financing, indicating that the high number of new enrollees did not lead to a corresponding increase in expenditures.

##### PhilHealth’s initial thrust: population coverage

In 1996, the Philippines introduced a program for poor households (the Sponsored Program), integrated in the National Health Insurance Program (NHIF). The expectation was that the LGUs would identify, enroll and pay (with support from national government) for the poor. A key design fault lay in the fact that LGUs were supposed to pay premiums (the level of which was defined by a sliding scale depending on the wealth ranking of the LGU) to PhilHealth for ‘poor’ households (as defined by the mayor) *and* they were still expected to finance their own health facilities. Although the national government rectified this and enrolled poor households using national government subsidies, it was not sustainable as the money did not come from a regular budget but from third-party sources (e.g. the Philippines Charity Sweepstakes). Since 2013, the national government has provided full subsidies, financed from the Sin Tax, and the 2017 national budget set aside PHP 3 billion (60 million USD) for having all Filipinos covered by PhilHealth [10].

At present, about 93 million of a total population of 104 million are covered by PhilHealth (Table 1), with the exact composition of the remaining 11 million non-covered not being known. Whereas SSS (the National Social Security System, the pension scheme) collects contributions from about 20 million employees (including the voluntarily insured), PHIC does so only from 14.6 million employees. As PhilHealth is a social insurance scheme, dependents (i.e. family members) of primary members are automatically enrolled once the member has started to pay his/her contribution.

**Table 1.** Membership structure of PhilHealth.

Catego-ries		Members	Dependents	Total	% of all members	Dependents/Member
Formal	Private	12.465.283	10.839.327	23.304.610	25,0	0,87
	Government	2.102.361	3.783.503	5.885.864	6,3	1,80
	Other formal	68.544	51.273	119.817	0,1	0,75
Informal	Migrant worker	659.311	951.543	1.610.854	1,7	1,44
	Informal worker	2.177.414	3.349.326	5.526.740	5,9	1,54
	Self-earning	409.751	586.676	996.427	1,1	1,43
	Other informal	14.335	19.855	34.190	0,0	1,39
Indigent		14.641.685	28.844.119	43.485.804	46,6	1,97
Sponsored		1.217.941	1.560.458	2.778.399	3,0	1,28
Lifetime		1.229.641	854.183	2.083.824	2,2	0,69
Senior Citizens		6.245.583	1.328.749	7.574.332	8,1	0,21
**All Members**		**41.231.849**	**52.169.012**	**93.400.861**		**1,27**

Members: those contributing to PhilHealth; dependents: those being automatically covered because a member did contribute

Source: PhilHealth Corporate Planning

##### Financial risk protection

Financial risk protection is a major goal of UHC. To assess whether this goal has been achieved equitably, two criteria are commonly used: the share of OOP of total health care expenses and the incidence of catastrophic health care spending at the household level. We look at the overall financial impact of PhilHealth and its effect on household-level spending.

Overall, there has been a massive growth in PhilHealth payments: from 17.1 billion PHP (342 million USD; 2006) to 101.8 billion PHP (2 billion USD; 2016) resulting in a Compound Annual Growth Rate (CAGR) of 19.5%, partly due to the doubling of the annual premium subsidy payments for poor households by national government. But population growth (1.8%), inflation (4.7%) and the general development of total health expenditures (11%) have mostly counterbalanced this growth, so the share of PHIC in THE rose only from 11% to 14%. The share of PhilHealth in total public health spending stands at 45% with the rest coming from national and local government (including Philippine Armed Forces and Police) and the SSS. Private spending accounts for 66% with the vast majority (about 82%) coming from OOP spending (Philippine National Health Expenditure Data 2014).

OOP payments have remained above 50% of total health care spending in the last 10 years and have, if anything, gone up rather than down. Almost half of the OOP spending (49.7%) goes to pharmaceutical products, followed by professional services (34.5%) and hospital services (15.8%) [16]. Lam and Rivera [17], in contrast, report that PhilHealth utilization among people admitted for illness was not significantly different among the poorest to richer quintiles (just below 50%, only the richest quintile standing out with a utilization rate of just under 60% [17], p. 14) and that PhilHealth coverage was associated with decreased occurrence of financial catastrophe in those receiving a hospital bill (46.5% vs. 58.1% [17], p. 16). A key indicator used by PhilHealth to measure the financial protection of its insurees is the so-called ‘support value’, i.e. the percentage covered by PhilHealth of the total cost incurred during a hospital stay, which has hovered between 50% and 60% in the years 2012 to 2015 (PhilHealth Stats and Charts 2012–2015).

Tobe and colleagues [18] present data that show how varied the support value can be, depending on the ownership and level of hospital (support level ranging from 22% to 45%), severity of case (43–60%), and type of membership (38–52%), leading to an effective support value for the individual that ranges from below 10% to 100%, and an assessment in 2012 of the implementation of no-balance-billing (introduced in 2011) found that Sponsored Program members were still being charged [11]. Lam and Rivera [17], in their review of PhilHealth performance, state:

Our small sample of NBB patients already documented violations in the practice where patients had to pay for services outside the hospital despite being admitted such as buying medicines or having tests. The result is that the effectiveness of the policy is diminished and the risk of financial catastrophe persisted.

Tobe and colleagues [18] conclude that ‘the reason for the existing gap between actual charges and the benefit ceilings is primarily because hospitals and doctors can decide their own fee schedules and PhilHealth has no mechanism to control this’.

PhilHealth has recently decided to shift the provider payment mechanism away from a fee-for-service system with benefit ceilings to case-based payment. PhilHealth started case-based payment (Philippines Diagnosis-Related Groups, PDRG) in 2009 and this was extended to another 21 case types (mostly among the 20 most frequently occurring diseases) in 2011 [18], with the goals of streamlining claims payments, increase in transparency, optimization of health service delivery and ultimately achieving greater financial protection for the corporation’s enrollees [19].

The so-called ‘Z-Benefits’ were introduced in June 2012 in response to calls for a ‘catastrophic fund’ and with the aim to cover particularly costly procedures and provide financial risk protection. The benefit package (with defined ‘package rates’) covers major forms of cancer (breast, prostate, cervical, colon), kidney transplantation, selected heart operations and orthopedic implants as well as care for premature and small newborns. In 2014, Z-Benefits were paid out in 2031 instances with a total value of 697 million PHP (14 million USD) – about 0.7% of all benefits paid (PhilHealth Stats and Charts, 2015).

So far, no data is available to assess whether the goals of these commendable PhilHealth initiatives have been achieved. At a more general level, Bredenkamp and Buisman [20] report that OOP health spending in the Philippines increased by 150% (real) from 2000 to 2012, mainly due to spending on medicines. As a result, the percentage of people incurring catastrophic payments has tripled (from 2.5% in 2000 to 7.7% in 2012) and in 2012, OOP spending on health pushed more than 1.5 million people into poverty.

#### (II) The role and impact of PhilHealth in service quality and utilization

The Philippine health care system is characterized by a combination of public and private ownership of assets and services. There are wide regional disparities in private and public hospitals in the country, ranging from a 82% private – 18% public mix in Region XI to 28% private – 72% public mix in Region VI [6]. This is per se not a problem, as there is no convincing evidence that private services differ from public services in terms of efficiency [12, p. 60]. The challenge is to balance general ease of access with equitable access, i.e. working towards access independent of socio-economic status, place of residence, cultural preferences, and ability to pay.

With the devolution of primary and secondary health services to LGUs in 1992, the administrative, operational and financial burdens of devolved health facilities and programs were turned over to LGUs, while upper-level hospitals remained under the Department of Health. This has led to a complex triangle of coordination, responsibilities and governance. While the DOH provides services, formally supervises and at the same time receives reimbursements from PhilHealth, the LGUs also provide services, receive reimbursements from PhilHealth and are crucial in helping PhilHealth to achieve high coverage rates throughout the provinces [4,6].

##### Equitable utilization of services

**Figure 4.**
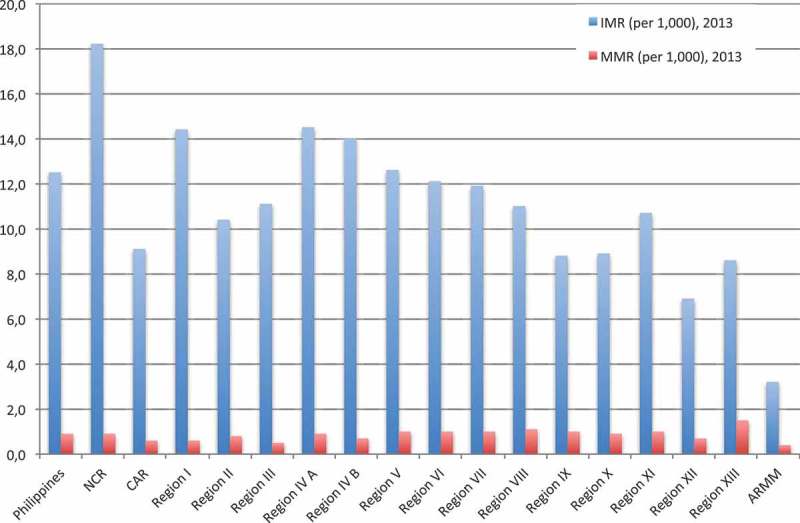
Geographical variation in infant and maternal mortality rate (2013). Source: Ref [46].

As a starting point, Figure 4 shows the substantial geographical differences in key health indicators such as life expectancy and infant mortality rate [11].

The UN Committee on Economic, Social and Cultural Rights (ICESCR, General Comment No. 14, 2000) has interpreted the right to health as being comprised of the following essential elements and principles:

*Availability*. Functioning public health and health-care facilities, goods and services, as well as programs, have to be available in sufficient quantity.

*Accessibility*. Health facilities, goods and services have to be accessible to everyone without discrimination. This has several dimensions, notably non-discrimination, physical accessibility and economic accessibility (affordability).

*Acceptability*. All health facilities, goods and services must be respectful of medical ethics and culturally appropriate.

*Quality*. As well as being culturally acceptable, health facilities, goods and services must also be scientifically and medically appropriate and of good quality.

Based on these elements and principles, the available literature primarily looks at equitable access and utilization of care, as these two aspects essentially capture how people react to the available services.

Although access and utilization of health care is only one of several factors contributing to these differences, it would be reasonable to argue that at least the health care provided (or rather not provided) should not lead to even larger disparities. Household utilization of these services is still largely dictated by the ability to pay. Poor households have been the lowest users of health services, with poor regions and the poorest income quintiles lagging behind in key interventions such as facility-based deliveries and prenatal care [21]. Table 2 below shows how both premiums paid and benefits received differ substantially between membership groups. The private sector (voluntary enrollment) receives per capita only about half of what each beneficiary pays (749 PHP benefits vs. 1649 PHP premium) and from a purely actuarial point of view the ratio of premiums paid to benefits received in the Individually Paying Program (IPP) indicates adverse selection (2968 PHP benefits vs. 587 PHP premium). However, from a public program/UHC perspective, such a cross-subsidization, i.e. transferring resources from the healthier to the less healthier segments of the population, can be viewed as the embodiment of risk sharing and solidarity.

**Table 2.** Beneficiaries, premiums collected, benefits paid per membership group (2016).

	Beneficiaries	Premiums collected (m)	Benefits paid (m)	Premium/beneficiary	Benefit/beneficiary
Private	23,424,427	38,615	17,543	1649	749
Government	5,885,864	9284	7424	1577	1261
Individually paying	6,557,357	3852	19,462	587	2968
Sponsored/Indigent	46,264,203	37,877	30,913	819	668
OFW	1,610,854	1076	1303	668	809
Senior citizens	9,658,156	13,045	25,107	1351	2600
**TOTAL**	**93,400,861**	**103,750**	**101,753**	**1111**	**1089**

m: million, IPP informal sector without migrant workers, Senior citizens: including lifetime members

Source: PhilHealth Corporate Planning

An older study from the Philippines [22] looked at the use of insurance in children under 5 years of age and found an overall under-utilization of about 15%, in particular associated with less-educated mothers and beneficiaries with shorter length of stay. These findings were corroborated by Faraon and colleagues [23], who showed the effect of gender, income status and type of membership (probably reflecting socio-economic characteristics) on frequency of use, and reported the lack of knowledge on how to file claims and on the benefit catalogue as significant predictors of under-utilization.

The Philippines face an epidemic of non-communicable diseases, chief amongst them diabetes, hypertension, cardiovascular disease and chronic obstructive pulmonary disease. Higuchi [24] reports that many patients took intermittent medication, largely due to their financial constraints.

A key strategy in further improving maternal and child health is the promotion of facility-based delivery. Gouda and colleagues [25] show that the likelihood of facility-based delivery for women who are insured is between 5% to 10% higher than for those without insurance, with a more pronounced impact amongst rural and poor women. Rivera [26], however, reports on surveys by the National Anti-Poverty Commission (NAPC) in 2015, which found that 4 in every 10 respondents were not aware of the PhilHealth benefits for pregnant mothers and their infants, and those who knew were discouraged from using their PhilHealth card due to administrative requirements by the clinic. The indirect cost of health care (e.g. transportation costs and lost wages) was another reason why poor families forewent their PhilHealth benefits. Mothers who used their PhilHealth card for childbirth still spent on average a total cash-out of 2275 PHP (45 USD) from their own pocket to pay for medicines and supplies, plus, if employed, they would lose the income of a day’s work. This needs to be put in relation to the average daily wage for this particular group of mothers, which was reported to be 481 PHP (9.50 USD).

##### Primary care

PhilHealth began financing a narrow range of outpatient services. TB DOTS, a Maternal Care Package, treatment of Rabies, Malaria, and HIV/AIDS were reimbursed at fixed rates, and an annual payment of 500 PHP (10 USD) was given to LGUs as ‘capitation’ for each family of the sponsored members and special groups (like teachers with the Department for Education) that the LGU or the National Government enrolled as members.

Availability and quality of services vary considerably; in particular, an urban–rural divide is clearly visible [27]. Thus, PhilHealth has been working to have the private sector involved in both primary and secondary care [21]. In April 2012, PhilHealth repackaged this outpatient benefits package and renamed it Primary Care Benefits (PCB), with the primary aim to incentivize the delivery of the package at the RHU level. Moreover, the PCB aims to involve the private sector with PCB I (covering doctors and diagnostics) and PCB II (covering outpatient pharmaceuticals). PCB II began with diabetes mellitus and hypertension and used an innovative voucher scheme, and all accredited primary care providers and drug outlets could participate.

A stocktaking in 2013 [28] clearly recommended the financing of a comprehensive set of outpatient care in order to improve health outcomes and reduce out-of-pocket spending. In response, PHIC explored the feasibility and impact of a program called Tsekap (Tamang Serbisyo para sa Kalusugan ng Pilipino; Proper Service for Filipino Health) with the idea to put a focus of the benefit package on those conditions and interventions that have a high burden of disease and a high cost-effectiveness ratio as well as drug benefits based on the clinical practice guidelines adopted by PhilHealth. Providers are both public and private, and the payment method for the package is reimbursements to drug outlets for the medicines, and capitation for health care providers for the Tsekap services including diagnostic tests. The Tsekap benefit package is estimated to cost 615 PHP (12 USD) per person or 1562 PHP (30 USD) per family at family size of 2.54 [29].

There is now a performance-based element in the management of Primary Care Benefits and simple verification mechanisms before the municipality receives the financing for each household. Many LGUs, however, did not comply with PhilHealth requirements to establish the necessary capitation trust fund (ensuring that the money would go exclusively to its health facilities), to formally enroll all poor households with an RHU and to document services rendered as well as the eligibility status of patients. As a result, PHIC payments (the primary care capitation) to LGUs have gone down dramatically because of unmet requirements stipulated by the corporation [11].

##### Secondary and tertiary care

Secondary and tertiary care is usually provided in hospitals, has only a very small (if any) preventive care component and covers a wide range of services from essential services (like Caesarian sections and basic surgery) to advanced high-technology and costly interventions like dialysis and cancer care. Such services are far less standardized, include greater discretionary power from the supplier side and are thus more difficult to control by the payer. In order to achieve allocative and technical efficiency, major challenges are controlling prices and volume. In addition, the payer should set the right framework and incentives to stimulate provider striving for improved quality [12, chapter 4].

As already mentioned above, PhilHealth still does not control the prices of services delivered at secondary and tertiary care level. The no-balance-billing policy has been implemented for government hospitals, but not fully complied with. In the private sector, the NBB was only introduced for some specifically contracted private hospitals (those delivering the Z-benefits, covering particularly high-cost interventions).

The case-based reimbursement rates (DRGs) are based on historical spending data and have not been updated since 2010, and are thus likely to inadequately reflect cost incurred in performing services and thereby counteracting the NBB policy. Since the introduction of the DRGs, no utilization review has been conducted. Fraudulent reimbursement claims (conservatively estimated by PhilHealth staff as being at least 10% of all claims) are rarely detected and, if they are, often do not have any consequences. The PhilHealth legal department is in the process of tightening processes here and expects to substantially increase the rate of detected fraudulent claims, and legal cases have been filed and penalties imposed.

At the DOH, the identification of drugs for listing in the Philippine National Formulary System (the former Philippine National Drug Formulary, PNDF) undergoes a defined process, but this is not strongly linked to how public health programs select their products. Furthermore, in 2014 the DOH developed the Drug Price Reference Index, but again this is slowly translating into changes in the way PhilHealth calculates its reimbursement rates (at present only for the newly costed packages and PhilHealth usually marking up a certain percentage on the DPRI).

Control of volume has so far not been in the focus of PhilHealth because there is mostly a perception of unmet needs and underserved populations. Nevertheless, there are signs of overprovision of specific services (see the following section) which might require intervention.

The revised Implementing Rules and Regulations of the National Health Insurance Act of 2013 (RA 7875) allow for the implementation of quality assurance standards (under Title V Quality Assurance and Accreditation), one of which is clinical practice guidelines (CPGs). In 2016, PhilHealth has started issuing such CPGs for acute gastroenteritis, urinary tract infection and community-acquired pneumonia (PhilHealth Circulars 2016–001 to −003). The Universal Health Coverage bill passed by the House of Representatives makes quality assurance mandatory for providers employing a comprehensive IT system (chapter X), and tasks the DOH to develop a competitive compensation package for medical staff

Peabody and colleagues [29] show that even modest financial incentives provided by a hospital-wide expansion of PhilHealth benefits (greater revenue in the form of insurance benefits covering 100% of costs for ordinary cases of common conditions such as pneumonia and diarrhea) led to significant and lasting positive effects on the quality of care provided. Although positive from a clinical quality perspective, this expansion also indicates an example of inefficient and non-strategic purchasing, as such common conditions should rather not been provided (and paid for) at hospitals, but at primary health centers. The same researchers [30] also found positive effects of direct pay-for-performance bonuses and, in a follow-up study, that the intervention sites continued to have significantly higher quality compared with the control sites with a very low (less than 1% per year) attrition rate in quality scores [31].

##### Strategic purchasing and the importance of the benefit package

Historically, PhilHealth has struggled from lacking a clear process of defining and expanding (or reducing) its benefits. So far, there has been no process for systematic updating of the benefit package, and additional benefits have been included incrementally and on an ad hoc basis [32] with political influences or lobbying from particular stakeholders being important drivers in the development of the benefit package [4] and, despite some recent initiatives like the Tsekap, overall the package is not geared towards tackling the country’s burden of disease, improving health outcomes, responding to the needs of the population or enhancing financial risk protection [32].

The current PhilHealth benefit package is broad (with the multitude of different packages sometime being confusing for members) and payment is mostly based on clinical inputs, not on diseases/conditions to be treated. In general, one can distinguish between an inpatient benefit package, an outpatient benefit package and special packages. Furthermore, PhilHealth has added specific benefits for the Sponsored Program that are not available to other NHIP beneficiaries. There are explicit limits on coverage such as non-prescription drugs and devices, alcohol abuse or dependency treatment, cosmetic surgery, optometric services, fourth and subsequent normal obstetrical deliveries, and cost-ineffective procedures as being defined by the corporation.

**Figure 5.**
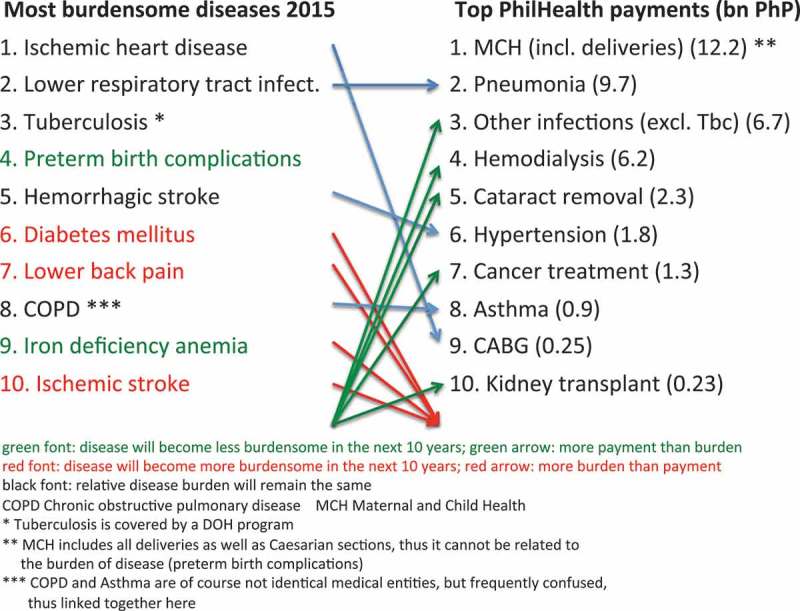
Burden of disease vis-à-vis PhilHealth top 20 payments plus Z-Benefits. Source: Authors, based on Ref [33] and PhilHealth Stats and Charts 2015.

If one puts the major contributors of the burden of disease vis-à-vis the top 20 diseases and Z-Benefits PhilHealth paid for, a serious imbalance can be observed (see Figure 5).

Four of the 10 most burdensome diseases are not in the top payment list at all and the most relevant illness (ischemic heart disease) is only covered by providing coronary artery bypass grafts for 458 patients nationwide. On the other hand, five of the top payment categories are not listed as most burdensome entities. Although cost-effectiveness needs to be considered when defining benefits, highly prevalent diseases and conditions, for which cost-effective interventions are available, would require reconsideration of how to spend PhilHealth resources.

A study by John Wong [33] looked at the diseases comprising the top 80% of disability-adjusted life years (DALYs) in the Philippines from 2015 to 2035 and came up with a list of the 48 most burdensome diseases coupled with cost-effective interventions that should ideally be covered, but this list does not, as the author points out, account for equity. This is covered by an additional list of 68 diseases to be considered for prioritization due to their disproportionate burden on vulnerable populations.

Currently, processes resembling Health Technology Assessment (HTA) are limited to drugs via the Formulary Executive Committee (FEC) at the DOH’s pharmaceutical division and the Z-benefits at PHIC. A major support in developing the benefit package can be expected with the Universal Health Coverage bill, as it explicitly mentions the use of HTA in prioritizing services and provides detailed conceptual and procedural guidelines.

## Discussion

The PhilHealth experience shows the need both to understand history and local environment, and to use a clear technical framework when analyzing and assessing key drivers and impediments towards achieving UHC. The role of external advice and support should be mentioned here. Between 2000 and 2016 a total of 99 externally funded projects were initiated. The majority of these (80) took place between 2000 and 2008 with a total volume of 4.1 million USD (PhilHealth International and Local Operations Department). Relating this spending to overall PhilHealth premium collection between 2000 and 2008 of 155 billion PHP (= 3.1 billion USD; PhilHealth Stats and Charts 2008) gives a ratio of about 0,13%. Although difficult to quantify, interview partners from PhilHealth, the DOH and international aid agencies indicated that this support was mostly useful, needed and overall had a clearly positive impact on the development and technical capacity of the organization.

### (I) The role and impact of PhilHealth in financing health care

The stipulated rise in PhilHealth contribution rates by a yearly 0.25% for the next 10 years (from currently 2.5% to 5.0% of earnings/income) should ideally be accompanied by an initiative to provide value for money to those who primarily fund PhilHealth: the formal sector. During interviews at PhilHealth and the DOH, as well as with providers, it transpired that the corporation primarily caters for the poor (which, from a UHC perspective, it should do), but somewhat neglects the needs of the middle- and high-income contributors such as paying for drugs for chronic conditions or improved financial protection. Ensuring that all PhilHealth members avail themselves of the services they need and also achieve better value for money could be done by, for example, expanding the full PCB to all members and to control balance billing for the non-indigent. Moreover, given that drugs are the major driver for the still high OOP expenses and the incidence of catastrophic health expenditures, PhilHealth may want to look in more detail at how it could support the purchase of outpatient drugs for its members as a crucial next step towards UHC.

Currently, PhilHealth covers about 93 million of the 104 million Filipinos. Amongst the 11 million non-covered are the near-poor, non-registered formal sector workers, self-employed professionals, and prison inmates. Given the importance of the formal sector in funding operations, improving revenue collection is crucial, e.g. via collecting agents and an improved database.

The changes introduced by the Bill would make the need for targeting and enrolling poor families obsolete. The move towards universal entitlement will avoid the ‘enrolling the informal sector’ issue, which has never been satisfactorily addressed, and move on to actually delivering health services. Reaching out to the non-poor informal sector to inform about rights and improve effective access to services and financial protection will remain difficult and a multitude of (ideally complementary) approaches might have to be employed here.

Research suggests that even at low levels of public spending, countries can make significant steps towards UHC [34]. Revenue raising needs to be coupled with the pooling of funds and the purchase of health services; it is the combination of reforms which drives improvements in health system performance [35]. Fiscal space for health analyses would explicitly take into account budget cycle planning and would account for political economy considerations. The Sin Tax has been widely commented on [9,36] and puts the Philippines into the still exclusive league of countries that have introduced sustainable alternative financing sources [37]. PhilHealth and all other government spending account for only about one-third of all health expenditures; thus public health expenditure stands at only 1.5% of GDP (compared to the Organisation for Economic Co-operation and Development [OECD] average of about 5.5% and Thailand at about 3.2%). While PhilHealth, as a single purchaser, could have had a more direct impact on service delivery, it has not yet flexed its muscles in pushing for more accountability, quality and price transparency [4]. It would substantially improve the corporation’s bargaining power and clout in defining quality and prices, were it to manage all the combined government funds. Such pooling, however, would face serious political barriers as it would entail a shift in power and control over funds. At any rate, the limited financial impact of PhilHealth remains a major challenge.

### (II) The role and impact of PhilHealth in service quality and utilization

Historically, both the public and the private sector have provided health insurance in the Philippines. PhilHealth from its beginning aimed at wide coverage and low-cost services, and thus upper- and middle-class Filipinos who wanted higher-quality benefit packages (and who could afford it) used private insurers and private services. This meant that the majority of those receiving services from the government-owned institutions were the poor. This entails the risk of ‘poor services for poor people’, although a clear difference between the sectors in terms of quality or efficiency cannot be shown [38].

The discussion about reducing financial burden and improving access and quality of care is strongly skewed towards the poorer parts of the population – but although overall spending is tilted towards the higher income quintiles, at the level of PhilHealth it is the formal sector which essentially cross-subsidizes the other members (see Table 2). While the country’s UHC agenda is laudable for its focus on the poor, other PhilHealth members must be kept in mind and must feel the benefits of being a member of PhilHealth for its political support and sustainability. In this regard, PhilHealth and the accredited hospitals need further strengthening to increase patients’ access to and utilization of quality health services and establish transparent and fair provider payment processes [39].

The development of an evidence-based benefit catalogue remains an ongoing process. There are clearly success stories as in the case of end-stage renal disease, where PhilHealth (not least due to special interest and political lobbying) provides comprehensive coverage (PhilHealth Stats and Charts 2016). This is in line with the international debate that suggests a much stronger effort in securing access to life-saving dialysis therapy [40]. Nevertheless, popular perception often links PhilHealth with narrow benefits, mainly focused on inpatient care, and many members seldom experience the usefulness of PhilHealth. Hospitalization is rare and, because of this rarity, health insurance usually ranks low in a household’s set of preferences [27]. For a more systematic approach, it might be advisable to start with the identification of new and emerging technologies and the development of explicit criteria that match policy priorities and enrollee demands. The institutionalized use of an analytic and evidence-based approach would eventually lead to a cost-effective technology assessment [41].

Ideally, PhilHealth, as the main purchaser of health services, should hold all the facilities accountable for the delivery and quality of care they provide, thus – at least partially – counterbalancing the negative effects of decentralization in the Philippines. The experience with a performance-based element in the management of Primary Care Benefits (see above the section on Primary Care) indicates that PhilHealth as a demand-side mechanism has not yet had a uniformly positive impact in reaching people and ensuring service and cost coverage in different parts of the country independent of an individual LGU’s priorities.

Conversely, facilities need to be technically and managerially competent and empowered to respond to such a strong purchaser. The role of the DOH will also need to be reviewed, since LGUs do not usually have the technical capacity and will need technical support from the DOH. LGU ownership with autonomy at the facility level may be an important structural aspect here.

## Conclusion

There is no UHC without a basic sense of solidarity in a society. When PhilHealth was created in 1995, the pressure to provide sufficient care for all Filipinos was less strong than it is now. The discussion about the right to health care and the development of the UHC concept has helped to move the spirit of solidarity forward over time. Clearly, the debate has moved from instrumentalizing PhilHealth in a political agenda and winning short-term electoral support to the inclusion of the poor and near-poor people as equal members of society in a social system.

The role social health insurance can play in working towards UHC is a contentious one. Any form of ‘contribution-based’ system has serious limitations in informal economies and a pathway of incremental population coverage might take decades. The concept of UHC goes beyond such gradual increases in coverage, and for NHI and UHC to fit comfortably together there needs to be a large increase in government budget transfers to cover payments from citizens unable to contribute. This could be linked to shifting away from the idea of contribution leading to entitlement, and towards the idea of citizenship leading to entitlement, i.e. establishing guaranteed access to a set of essential services. The current Philippine UHC Bill does exactly this and can be seen as a paradigmatic shift in thinking about the role of an NHI in contributing to UHC.

Although not (yet) successful in managing a larger share of THE, the corporation has been working on its internal processes as well as its relation with providers, resulting in numerous quality and financial protection initiatives, the results of which might be fully felt only in the years to come.

We identified three potential avenues for further debate and inquiry:

(i) Improving PhilHealth governance
(ii) Increasing financial impact
(iii) Strategic purchasing based on a rational benefit package and improved provider payment

### (i) Improving PhilHealth governance

Although the Universal Health Coverage Bill stipulates a representative of the LGUs, thus providing a formal link, the relation between PhilHealth and the DOH is not specified. Furthermore, the president and CEO of PhilHealth is supposed to have only a one-year tenure – not enough for developing and executing a strategic perspective.

A major challenge for advanced provider management and strategic purchasing in the Philippines lies in the highly decentralized health sector context with local government units having a prominent role. Implementation of rigorous purchasing arrangements is constrained by the complex and interwoven governance and accountability arrangements (or the lack thereof) between the DOH, PhilHealth and the LGUs [11]. Participation of enrollees and citizens in the benefits package and other decision-making of PhilHealth can also improve accountability and political acceptability by incorporating their (social) values in health insurance policy [41].

Finally, the corporation may be well advised to establish some form of internal evaluation process, which would look at the successes and failures of initiatives, and draw lessons from this for future planning and projects. This could be done by an executive department, possibly reporting directly to the board and/or independent evaluators who would be hired for providing an external and unbiased view.

### (ii) Increasing financial impact

There are good arguments for combining the various strands of government-financed individual health care (e.g. DOH, LGUs, Armed Forces of the Philippines, Department of Education and Philippine Charity Sweepstakes Office, amongst others) under the umbrella of PhilHealth – this would lead to total of about one-third of THE and would constitute a significant financing source, which could effectively influence health policy and provider behavior. This, however, might – for reasons of power and control – be difficult to achieve, as many countries are stuck with a ‘mixed landscape’ of (i) a separate purchasing agency through which payroll contributions and some budget transfers flow, and (ii) continued direct budget funding by the DOH.

The gradual rise from 2.5% to 5.0% contribution of gross income to PhilHealth is politically contentious and PhilHealth should try to show tangible effects for the middle- and higher-income members. The rise in minimum contribution for the IPP from 1200 PHP to 2400 PHP (24 to 48 USD) in 2013 was met with substantial resistance, protests and street rallies. Nevertheless, if PhilHealth is supposed to play a major role in controlling prices and cost escalation in the private sector, more money needs to be channeled through this payer system coupled with appropriate governance and regulation.

### (iii) Strategic purchasing based on a systematic process of defining benefits and improved provider payment

Up until this point, PhilHealth has mostly made ad hoc decisions about what packages would be covered under the National Health Insurance (now: Security) Program and there was no clear and definite decision-making process. A systematic approach is needed in order to assess the effect of coverage decisions on different strata of the population [42]. Such a systematic process to work through the many trade-offs of efficiency with equity, financial protection, burden of disease, etc. would also allow PhilHealth to manage requests for inclusion by referring to a due process based on burden-of-disease and HTA data. The goal is a comprehensive Guaranteed Health Benefit Package (GHBP) for all PhilHealth members.

Worldwide, there is a strong movement to couple provider payments to some form of review and quality, which is especially important for the Philippines where private providers play a dominant role in service delivery. It might still be advisable to pursue this avenue further, albeit with due care [43, p. 117, p. 120], and linking it to the questions of public hospital autonomy, price control and monitoring co-payments.

PhilHealth might be well advised to rigorously push for eHealth in order to ‘leapfrog’ [44] key processes (drug management, payment, biometric data, eligibility testing), to scale-up data collection, to push for a Philippine Health Information Exchange Architecture (PHIEA) and to improve convenience for patients and providers [27].

At any rate, reforms need ‘to reflect an assessment and understanding of the policy reform environment *as it is*, including actual implementation conditions, constraints, and requirements’ [45, p. 4]. Talk of ‘comprehensive reform’, ‘fully integrated systems’ and ‘clear delineation of tasks and responsibilities’ (to name but a few) – as much as they are desirable – usually does not tally well with the politico-economic reality of health systems and their numerous stakeholders and partial interests. If the goals and aims are clear, a modest but continuous working at the margins and seizing windows of opportunity might be the recommended way forward.

## Paper context

It remains difficult to enroll those in informal employment and/or those who are poor into a national health insurance (NHI) scheme. The Philippines’ experience provides examples of improving health financing: (i) introducing a so-called ‘Sin Tax’ on tobacco and alcohol and (ii) currently discussing a law which would make every Filipino automatically an NHI member. Challenges for the NHI are its still low financial impact, and insufficient control of health care providers.
